# Mechanistic Insights Into the Inhibition of *Clostridioides difficile* Binary Toxin by Indolylmethyl Glucosinolate and Indole‐3‐Carbinol

**DOI:** 10.1155/sci5/8115947

**Published:** 2026-05-18

**Authors:** Ariful Islam, Sumaiya Jahan Supti, Faria Tasnim, Md. Zahid Hasan, Mst Naharina Nuryay, Nabida Tabassum, Maysha Fahmeda Priota, Md. Jan Sadur Rahman Moon, Taha Alqahtani, Magdi E. A. Zaki, Subir Sarker, Md. Eram Hosen

**Affiliations:** ^1^ Department of Genetic Engineering and Biotechnology, University of Rajshahi, Rajshahi, 6205, Bangladesh, ru.ac.bd; ^2^ State Key Laboratory of Microbiology and Bioinformatics, Department of Microbiology, Shaheed Shamsuzzoha Institute of Biosciences, Affiliated With University of Rajshahi, Rajshahi, Bangladesh; ^3^ Department of Microbiology, Rajshahi Institute of Bioscience, Affiliated With University of Rajshahi, Rajshahi, 6205, Bangladesh; ^4^ Department of Chemistry, College of Science, Imam Mohammad Ibn Saud Islamic University (IMSIU), 11623, Riyadh, Kingdom of Saudi Arabia, imamu.edu.sa; ^5^ Biomedical Sciences and Molecular Biology, College of Medicine and Dentistry, James Cook University, Townsville, Queensland, 4811, Australia, health.qld.gov.au

**Keywords:** antibacterial activity, *Brassica oleracea*, CDTa toxin, *Clostridioides difficile*, molecular docking, molecular dynamics simulation

## Abstract

*Clostridioides difficile* infection (CDI) remains a significant healthcare challenge, primarily due to its toxin‐mediated pathogenesis that results in severe gastrointestinal complications. This study investigates the inhibitory potential of bioactive compounds derived from *Brassica oleracea* L., specifically targeting the CDTa subunit of the binary toxin, using both computational and experimental approaches. Molecular docking analysis identified indolylmethyl glucosinolate (−9.1 kcal/mol) and indole‐3‐carbinol (−8.75 kcal/mol) as top candidates, demonstrating high binding affinities to the CDTa protein. Stability and dynamic behavior of the ligand–protein complexes were further assessed through 100‐ns molecular dynamics simulations, which confirmed their stable interactions. Thermodynamic evaluations using MM‐GBSA calculations revealed favorable binding free energies, supporting their potential as effective inhibitors. In parallel, the antibacterial efficacy of these compounds was validated through in vitro antibacterial assays, where indolylmethyl glucosinolate and indole‐3‐carbinol exhibited maximum inhibition zones of 23.33 ± 0.67 mm and 22.67 ± 0.33 mm, respectively, at a concentration of 100 μg/mL. In MIC and MBC assays, both compounds showed significant antibacterial activity, with indolylmethyl glucosinolate demonstrating slightly higher potency (MIC: 10.33 ± 0.72 μg/mL; MBC: 23.33 ± 1.36 μg/mL) than indole‐3‐carbinol (MIC: 11.33 ± 0.27 μg/mL; MBC: 26.67 ± 3.60 μg/mL). Additionally, PCA and DCCM revealed distinct conformational stabilization patterns and correlated motion of CDTa upon ligand binding, supporting a potential antivirulence interaction. Moreover, ADMET analysis revealed differences in their pharmacokinetic and toxicity profiles, providing further insights into their therapeutic potential. Overall, these findings suggest that *B. oleracea*–derived compounds exhibit a direct antibacterial activity against *C. difficile* while also demonstrating computationally supported inhibition of CDTa, indicating a complementary antivirulence mechanism that may contribute to their therapeutic potential in CDI.

## 1. Introduction

The toxin‐producing, Gram‐positive anaerobic bacterium *C. difficile* (*C. difficile*) has emerged as an important driver of healthcare‐associated illnesses, especially antibiotic‐related diarrhea contracted in healthcare facilities [1]. Infection with *C. difficile* is associated with a spectrum of gastrointestinal disorders, ranging from mild diarrhea to life‐threatening conditions such as toxic megacolons, pseudomembranous colitis, and systemic inflammatory response syndrome [2]. *C. difficile* infection (CDI) poses considerable public health danger, as indicated by its inclusion in the Centers for Disease Control and Prevention (CDC) list of pathogens with an “urgent” threat level [3]. In the United States alone, CDI accounts for over 500,000 cases annually, resulting in approximately 29,000 deaths and incurring healthcare costs estimated between $1 and $3 billion [4]. CDI predominantly affects hospitalized patients and those receiving antibiotic treatment, disrupting the normal gut microbiota and facilitating the overgrowth of *C. difficile* [5]. Moreover, *C. difficile* spores can survive in healthcare settings and cause recurring infections because they are resistant to disinfectants, environmental factors, and antibiotics [6, 7].

**FIGURE 1:**
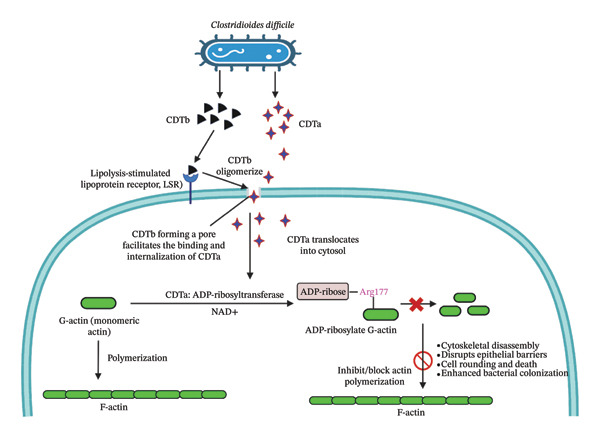
Mechanism of *Clostridioides difficile* toxin B (CDTb) and toxin A (CDTa) action in host cells: CDTb binds to the lipolysis‐stimulated lipoprotein receptor (LSR) and oligomerizes to form a pore, facilitating CDTa internalization. Upon translocation into the cytosol, CDTa exerts ADP‐ribosyltransferase activity, transferring ADP‐ribose from NAD+ to Arg177 of monomeric G‐actin. This modification inhibits actin polymerization, leading to cytoskeletal disassembly, epithelial barrier disruption, cell rounding, and death, ultimately promoting bacterial colonization.

*C. difficile* pathogenicity is primarily driven by its production of potent toxins, including the binary toxin *C. difficile* transferase (CDT), composed of two distinct subunits: CDTa (the enzymatic component) and CDTb (the binding component) [8]. Although only 5%–30% of clinical isolates produce CDT, its presence is strongly associated with increased morbidity, mortality, and more severe disease, especially in hypervirulent strains [9]. CDT adds an extra layer of complexity in CDI management [10]. The CDTb subunit first binds to the lipolysis‐stimulated lipoprotein receptor (LSR) on host cells and undergoes proteolytic cleavage, triggering oligomerization [11]; this facilitates the binding and entry of CDTa [12]. Once the toxin complex is internalized into an endosome, the acidic environment induces a conformational change in CDTb, allowing CDTa to translocate into the cytoplasm [13]. Inside the host cell, CDTa acts as an ADP‐ribosyltransferase, targeting monomeric G‐actin at arginine‐177 [14, 15]. This post‐translational modification prevents actin polymerization, leading to the breakdown of filamentous actin (F‐actin) structures [16]. The collapse of the cytoskeleton causes cell rounding, disruption of tight junctions, and increased epithelial permeability (Figure 1) [17, 18]. These effects compromise the intestinal barrier, promoting inflammation, tissue damage, and enhanced bacterial colonization [19]. Structurally, CDTa is composed of two domains joined by a flexible loop: the N‐terminal domain interacts with CDTb, while the C‐terminal domain carries out its toxic enzymatic function [15, 20]. Understanding the molecular mechanisms of CDTa’s interaction and enzymatic activity is crucial for the development of targeted therapeutics. Given its significant role in virulence and its correlation with severe clinical outcomes, CDTa represents a promising target for novel treatment strategies aimed at mitigating the effects of hypervirulent CDIs.

Commonly used antibiotics and medications for the treatment of CDI include fidaxomicin, vancomycin, and metronidazole [21]. However, these treatments are not without limitations. Metronidazole, previously recommended as a first‐line therapy for nonsevere CDI [22], has seen reduced efficacy in recent years and is now reserved for use when vancomycin or fidaxomicin is unsuitable [23]. Although vancomycin and fidaxomicin are FDA‐approved and exhibited greater efficacy, they remain associated with significant recurrence rates, ranging from 15% to 35% [21, 24]. Vancomycin, which has broad‐spectrum activity against Gram‐positive bacterial effects [25], is only partially absorbed into the bloodstream [26]. As a result, it can disrupt gut microbiota diversity, predisposing patients to recurrent infections [27]. Fidaxomicin offers greater selectivity for *C. difficile* and is associated with lower recurrence rates, but its widespread use is often limited by high treatment costs [28]. Emerging alternatives such as fecal microbiota transplantation (FMT) show promise in restoring gut microbial balance and lower recurrence rates. However, FMT is not without risks, including the potential for transmission of infectious agents, particularly in immunocompromised or elderly patients [29–32].

The limitations of current antibiotic treatments and the high recurrence rates have intensified efforts to develop novel therapeutic strategies against CDI. One promising avenue of research involves exploring natural compounds with antimicrobial properties [33]. As a member of the *B. oleracea* species, the Italica cultivar group includes broccoli, a nutritious green vegetable that belongs to the *Brassicaceae* family [34]. This vegetable is considered very important for health because it has many benefits, such as fighting bacteria, reducing oxidation, helping prevent cancer, supporting the immune system, controlling diabetes, protecting the liver and heart, and improving memory [35, 36]. It contains several bioactive phytochemicals, including selenium, indolylmethyl glucosinolate, sulforaphane, and polyphenols, which contribute to its health benefits [37–39].

Recent advances in biomedical research have facilitated the discovery of novel antimicrobial agents, including those derived from plant‐based sources [40, 41]. Building upon this foundation, the present study aims to identify potential phytochemical inhibitors of the CDTa protein from *C. difficile*, focusing on compounds found in broccoli. By targeting the enzymatic subunit of the binary toxin, this approach may offer a novel therapeutic pathway for reducing the severity and recurrence of CDI.

## 2. Materials and Methods

### 2.1. Mining and Preparation of *B. oleracea* Phytochemical Ligands

A set of 51 chemical compounds derived from *B. oleracea* was retrieved in SDF format from the PubChem database (https://pubchem.ncbi.nlm.nih.gov/). Subsequently, these compounds were subjected to energy optimization using Avogadro software (Version 1.2.0) with the MMFF94 force field. The data underwent meticulous refinement to ensure accuracy prior to further analysis [42].

### 2.2. Protein Preparation

The structural model for the CDTa subunit was obtained from the Protein Data Bank (PDB ID: 6X41) [43], which represents the enzymatically relevant domain of CDTa. While no associated peer‐reviewed publication or preprint is currently available for this structure, it provides a high‐resolution x‐ray diffraction model (2.36 Å) suitable for computational docking and molecular dynamics (MD) simulations. At first, Discovery Studio (Version 21.1.0.0) was used to remove any extraneous molecules. The cleaned protein structure was then optimized and energy minimized within the Swiss PDB Viewer program (Version 4.1) using the GROMOS96 43b1 force field [44].

### 2.3. Molecular Docking

Molecular docking was performed using proteins CDTa (PDB ID: 6X41) from *C. difficile* and plant‐derived chemicals from *B. oleracea*. This procedure made use of PyRx, a docking simulator based on the AutoDock Vina wizard [45] (https://sourceforge.net/projects/pyrx/), which was updated to Version 0.8. Modified versions of previous methods [46–48] were used to conduct the molecular docking. To begin, the data were cleaned up by transforming the plant ligands into PDBQT and the protein structures into a suitable macromolecule format. The CDTa protein (PDB: 6X41) does not have a cocrystallized ligand or defined binding site in its structure, so we used a blind docking approach to find possible binding sites all over the protein’s surface that could interact with the compounds we chose from *B. oleracea* and the control drug, ciprofloxacin. The coordinates *X*: −5.1080, *Y*: 20.4920, *Z*: −22.4990 were used to center the grid box, which had dimensions *X*: 79.2991, *Y*: 55.7939, and *Z*: 55.6731 (in Å). In the end, the docking simulation determined which compounds performed best by determining which ones had the lowest binding energies. A subsequent analysis was conducted utilizing the Discovery Studio software to determine the precise interactions and orientation of the bound plant chemicals to the protein. Molecular docking binding energies are reported in kcal/mol, consistent with the native output of the docking software.

### 2.4. MD

MD simulations were carried out utilizing the AMBER14 force field in YASARA Dynamics software (Version 19.12.4) [49, 50]. At first, we tuned the hydrogen bond network and polished the docked complexes. Next, the steepest gradient approach and the TIP3P water solvation model were used to minimize protein complexes [51]. The parameters used were density 0.997 g/L, temperature 25°C, and pressure 1 atm. Physiological conditions were maintained at 310 K, pH 7.4, and 0.9% NaCl concentration to neutralize the simulated system [52]. A simulation time step of 1.25 frames per second was utilized, and the particle mesh Ewald (PME) approach with a cutoff radius of 8.0 Å was employed to calculate long‐range electrostatic interactions [53]. The whole simulation lasted 100 ns, and data about the trajectory were saved every 100 ps [54]. Measurements such as root‐mean‐square deviation (RMSD), solvent‐accessible surface area (SASA), radius of gyration, root‐mean‐square fluctuation (RMSF), dynamic cross‐correlation matrix (DCCM), and hydrogen bond formation were included in the analysis of the simulation trajectory.

### 2.5. Calculation of Binding Free Energy With MM/PBSA

Determining the binding free energy is a crucial step in evaluating the potency of a drug–protein interaction. This method yields useful data regarding the stability and energetic characteristics of the drug–protein complex. The binding free energy was determined in YASARA software by evaluating several complex snapshots using the MM–Poisson–Boltzmann surface area (MM–PBSA) approach. Based on the following formula, the MM/PBSA binding free energy was calculated:

(1) MM/PBSA binding free energy=EpotReceptor+EsolvReceptor+EpotLigand+EsolvLigand−EpotComplex−EsolvComplex.

The AMBER14 force field was used for calculations, and YASARA macros were used to simplify the MM–PBSA binding energy estimate. MM/PBSA binding free energies are reported in kJ/mol, as generated by the MM/PBSA analysis workflow.

### 2.6. Principal Component Analysis (PCA)

To investigate the overall structural variability among the protein–ligand complexes including comparisons with both the apo protein and a reference drug‐bound complex, PCA was applied, taking into account all relevant structural descriptors. This dimensionality reduction technique facilitated the detection and classification of conformational changes that emerged throughout the MD simulations. PCA was conducted by constructing and diagonalizing the covariance matrix of atomic fluctuations, followed by solving the corresponding eigenvalue and eigenvector equations. The resulting eigenvalues quantified the extent of structural fluctuations, while the eigenvectors indicated the principal directions of motion. Prior to analysis, 100‐ns MD trajectories were standardized by mean‐centering and scaling to unit variance. The PCA calculations were carried out in Python (v3.11) using the Scikit‐learn library (v1.2), and the outcomes were visualized using Matplotlib (v3.7).

### 2.7. Absorption, distribution, metabolism, excretion, and toxicity (ADMET) Analysis

An ADMET study was carried out to evaluate the phytochemicals’ potential as lead compounds once the docking and dynamics simulations were finished. The pkCSM [55] and SwissADME [56] web services were used for this purpose, to evaluate ADMET profiles and to find which molecules adhered to Lipinski’s rule of five [57], respectively.

### 2.8. *In Vitro* Antibacterial Activity

#### 2.8.1. Chemicals and Reagents

The main lead compound of this study was indolylmethyl glucosinolate; however, the exact compound was not commercially available. Therefore, glucobrassicin potassium salt (3‐indolylmethyl glucosinolate potassium salt; empirical formula: C_16_H_19_KN_2_O_9_S_2_; CAS No.: 143231‐38‐3; molecular weight: 486 g/mol; HPLC grade; Sigma‐Aldrich, USA) was used as a substitute for in vitro validation experiments. Indole‐3‐carbinol (empirical formula: C_9_H_9_NO; CAS No.: 700‐06‐1; molecular weight: 147.17 g/mol; EC No.: 211‐836‐2; MDL No.: MFCD00005632; Catalog No.: I7256; Sigma‐Aldrich, USA) was employed as a separate reference compound. HPLC‐grade methanol (Sigma‐Aldrich, USA) was used for sample preparation. The antibiotic ciprofloxacin was provided by Square Pharmaceuticals Limited (Bangladesh). Luria Bertani (LB) broth and LB agar media were purchased from Sigma‐Aldrich (USA).

### 2.9. Collection of Bacterial Samples

*C. difficile* bacteria were sourced from Professor Joardar’s DNA and Chromosome Research Laboratory at the Department of Genetic Engineering and Biotechnology, University of Rajshahi, Bangladesh. These microbes were first isolated from CDI patients. Their overnight incubation at 37°C followed collection, and they were cultivated on LB agar media. Cold storage at −80°C was used for the long‐term preservation of *C. difficile*. The bacteria were handled and used in accordance with all applicable safety standards and regulations due to its classification as a Biosafety Level 2 (BSL‐2) pathogen.

### 2.10. *In Vitro* Antibacterial Activity Determination

Following computational analysis that suggested significant inhibitory potential against *C. difficile*, indolylmethyl glucosinolate and indole‐3‐carbinol were identified as the most promising compounds for further investigation of their antibacterial activity. Test solutions were prepared by dissolving the compounds in 60% methanol. The antibacterial assay was then conducted *in vitro* using a modified disc diffusion method, with concentrations of 50, 75, and 100 μg per disc. Bacterial cultures were incubated overnight at 37°C in nutrient broth, with constant shaking at 180 rpm. Following incubation, bacterial suspensions were prepared to a concentration of 1 × 10^6^ CFU/mL and spread evenly onto LB agar plates. Discs (5 mm diameter) of Whatman No. 1 filter paper were impregnated with 50, 75, or 100 μg of either indole‐3‐carbinol or indolylmethyl glucosinolate and placed on the agar surface. Ciprofloxacin served as the positive control to evaluate antibacterial effectiveness. After 24 h of incubation, the formation of clear inhibition zones around the discs was observed, indicating bacterial growth suppression. These zones were measured in millimeters (mm). The experiment was performed in triplicate to ensure reliability, and the results were expressed as mean values with standard deviations.

### 2.11. Determination of Minimum Inhibitory Concentration (MIC)

MIC determination: In this study, the tube dilution method was used to determine the MIC of the most effective indolylmethyl glucosinolate and indole‐3‐carbinol, in accordance with the Clinical and Laboratory Standards Institute (CLSI) guidelines with some modifications [58]. Dimethyl sulfoxide (DMSO) in sterile nutritional broth was used to prepare serial dilutions of the indolylmethyl glucosinolate and indole‐3‐carbinol, starting from a stock solution of 5 mg/mL each, at concentrations ranging from 1 to 15 μg/mL. Three rows of 15 sterile test tubes each were assigned to the control medication, ciprofloxacin, the indolylmethyl glucosinolate, and indole‐3‐carbinol. One milliliter of the sample concentration in nutrient broth and one hundred microliters of the tested bacteria were placed in each test tube. After that, the test tubes were incubated for 24 h at 37°C. The positive control was nutrient broth infected with ciprofloxacin, whereas the negative control was nutrient broth containing just bacteria.

### 2.12. Determination of Minimum Bactericidal Concentration (MBC)

The MBC was identified as the lowest concentration of indolylmethyl glucosinolate, indole‐3‐carbinol, and ciprofloxacin that eradicated 100% of the test organisms. The MBC was established by adding 50‐μL aliquots of the serial dilution that exhibited no visible growth after incubation in the MIC assay to 150 μL of broth in a test tube, followed by incubation for 48 h at 37°C. MBC exhibited the lowest concentrations of indolylmethyl glucosinolate, indole‐3‐carbinol, and ciprofloxacin. The term MBC endpoint refers to the concentration of an antimicrobial agent at which 100% of the bacterial population is eradicated [59].

### 2.13. Statistical Analysis

All values were provided as the mean ± SE after data were processed using MS Excel (Version 2016). In order to compare the mean of three replicates with a significance level of *p* < 0.05, the SAS program (Version 9.1.3) was utilized, along with Duncan’s multiple range test and one‐way ANOVA.

## 3. Results and Discussion

### 3.1. Molecular Docking Study

Molecular docking is an essential technique in computational structure–based drug discovery, utilized to predict the optimal orientation and binding of a small molecule within a protein’s active site. This approach plays a critical role in lead compound optimization and facilitates the rational design of molecules that enhance protein–ligand interactions, thereby improving therapeutic efficacy and reducing unwanted interactions [60, 61]. The regulation of virulence factor secretion by CDT proteins has been implicated in the pathogenesis of *C. difficile*, which is closely associated with CDI [62]. Despite this known connection, limited research has been conducted on the inhibitory effects of phytochemicals derived from *B. oleracea* on the CDTa protein. In the present study, the ligands were computationally predicted to bind effectively with the target protein, implying the potential to inhibit the biological activity of *C. difficile* toxin, a key player in the expression of virulence genes. Among the 51 *B. oleracea* compounds evaluated (Supporting Table S1), indolylmethyl glucosinolate and indole‐3‐carbinol stood out as leading candidates due to their favorable binding energies. A detailed summary of the docking results, including binding affinities and molecular interactions of these ligands with the 6X41 protein, is presented in Table 1.

**TABLE 1:** The interactions between the ligand molecules and the *Clostridium difficile* (CDTa) 6X41 protein, covering aspects such as binding energy, types of noncovalent interactions, participating amino acid residues, bond types, and interaction distances.

Complex	Binding energy (kcal/mole)	Amino acid residues	Bond types	Distance (Å)
6X41+ indolylmethyl glucosinolate	−9.1	A:ASN262	H	2.15
		A:ARG302	H	2.21
		A:ARG302	H	2.75
		A:ASN342	H	2.52
		A:ASN263	H	2.28
		A:ASN263	H	2.96
		A:PHE343	H	2.48
		A:ARG359	H	5.34
		A:GLN307	C‐H	3.51
		A:PHE345	P‐P	4.00
		A:PHE345	P‐P	4.71
		A:GLU308	AC	5.31

6X41+ indole‐3‐carbinol	−8.75	A:ALA31	H	1.85
		A:GLU35	H	4.77
		A:GLU47	H	4.92
		A:THR177	H	3.04
		A:LYS43	P‐A	5.00

6X41+ ciprofloxacin	−8.2	A:LYS24	H	2.27
		A:VAL182	H	1.95
		A:PHE101	CH	3.45
		A:PRO151	A	4.54
		A:SER147	PS	3.80
		A:ASP145	HA	3.17

*Note:* Ciprofloxacin served as a positive control for comparison. H: Hydrogen bond; C–H: carbon–hydrogen bond; P–P: pi–pi stacked bond; P–S: pi–sigma bond; P–An: pi–anion bond; P–H: pi–donor hydrogen bond; HA: halogen bond (fluorine).

Abbreviations: AC, attractive charge; P–A, Pi–alkyl.

The interaction of indolylmethyl glucosinolate with the 6X41 protein exhibited a binding energy of −9.1 kcal/mol and was mediated by a range of amino acid residues. Notably, the target–ligand complex interaction template showed seven hydrogen bond interactions with ASN262, ARG302, ASN342, ASN263, and PHE343 amino acid residues. Carbon–hydrogen and pi–pi stacking interactions were observed with GLN307 and PHE345, respectively, and pi–pi interactions were also noted at PHE345. The 6X41+ indole‐3‐carbinol complex evolved a binding energy of −8.75 kcal/mol (Table 1) and formed four hydrogen bond interactions with target protein at ALA31, GLU35, GLU47, and THR177 (Figure 2(a)). Only a single pi–alkyl interaction was identified at LYS43 (Figure 2(b)). Compared to the control compound ciprofloxacin, our top two compounds demonstrated stronger binding energies and more extensive interactions with the target protein. The control complex 6X41+ ciprofloxacin exhibited two hydrogen bonds with LYS24, VAL182, one carbon–hydrogen bonds with PHE101, one alkyl bond with PRO151, one pi–sigma bond with SER147, and one halogen bond with ASP145 (Figure 2(c)).

FIGURE 2The molecular docking interactions between the 6X41 protein of *Clostridium difficile* and two compounds derived from *Brassica oleracea*: (a) indolylmethyl glucosinolate and (b) indole‐3‐carbinol. The compounds are represented in both two‐dimensional format and surface view. (c) Ciprofloxacin was used as a reference compound for validation.

**Figure:**
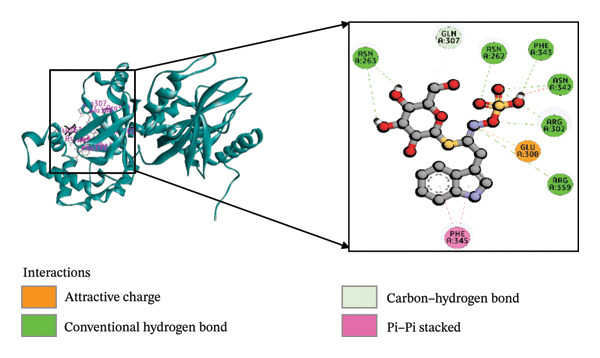
(a)

**Figure:**
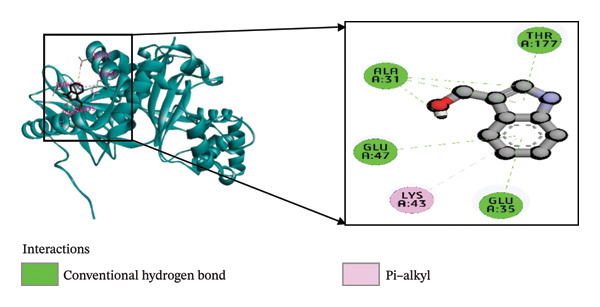
(b)

**Figure:**
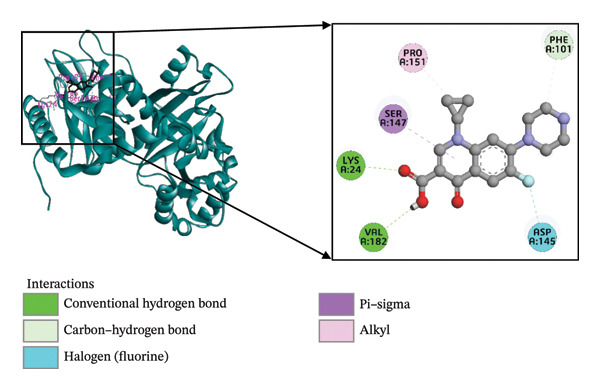
(c)

The 6X41 protein is still unexplored for inhibition by phytocompounds through molecular docking studies. These results suggest that the compounds may interact with potential binding sites on the 6X41 protein, implying that these phytochemicals could potentially inhibit the CDTa protein. Indolylmethyl glucosinolate and their hydrolysis products, such as isothiocyanates, exhibit antimicrobial activity by disrupting bacterial cell walls and inhibiting key bacterial enzymes [63]. Their ability to disrupt bacterial virulence mechanisms aligns with the findings of this docking study, where strong binding interactions were observed with the CDTa protein. Indole‐3‐carbinol, although primarily known for its anticancer properties, has also shown potential in modulating bacterial virulence factors [64]. However, its ability to inhibit bacterial proteins is still emerging, but the current docking results suggest that it may be capable of forming stable interactions with bacterial proteins involved in virulence.

Although molecular docking was performed using the isolated CDTa structure (PDB 6X41), published CDTb–CDTa cocomplex structures [13] demonstrate that the enzymatic active site of CDTa remains solvent‐accessible within the assembled binary toxin. This supports the structural plausibility of the selected docking target for ligand engagement in a biologically relevant context. Accordingly, the observed docking interactions are considered informative despite the absence of explicit protein–protein complex docking in the present study.

### 3.2. MD Simulation

The results from molecular docking were further corroborated using MD simulations, which confirmed that the ligands sustained comparable interactions in both docking and MD simulations. However, some alterations in the bond types for specific amino acids were observed (Supporting Figure S1 and Table S2). MD simulations were employed to analyze the atomic‐level behavior of protein–ligand complexes, providing critical insights into drug design, uncovering underlying biological mechanisms, and enhancing the understanding of structure–function relationships [65–67]. To evaluate the stability and rigidity of the most promising compounds, MD simulations were performed over a 100‐nanosecond (ns) timeframe, enabling the identification of potential inhibitors. The stability of the complexes was assessed using various metrics, including RMSD, SASA, radius of gyration (Rg), hydrogen bonding, and RMSF. The evaluation primarily focused on the CDTa protein complexes (PDB ID: 6X41), as shown in Figure 3.

FIGURE 3Analysis of (a) RMSD and (b) radius of gyration of complexes between indolylmethyl glucosinolate, indole‐3‐carbinol, and target protein of *Clostridioides difficile* toxin CDTa (PDB: 6X41) at 100‐ns molecular dynamics simulation.

**Figure:**
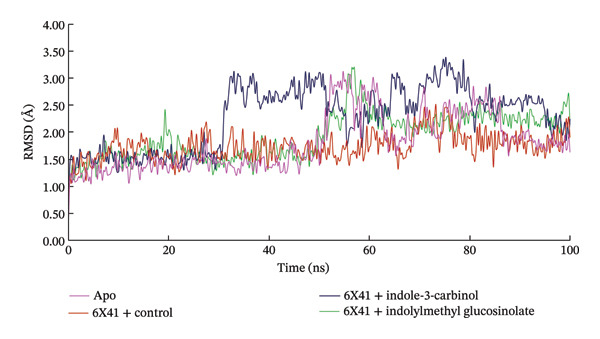
(a)

**Figure:**
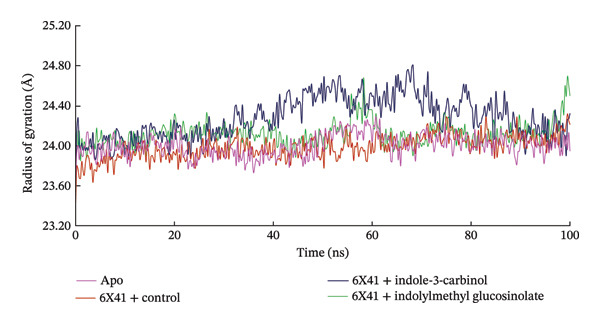
(b)

### 3.3. Analysis of RMSD

RMSD measures the average displacement of atoms relative to a reference structure over a specified period [68]. In the current research, a 100‐ns simulation was used to track the RMSD of the CDTa protein’s Cα backbone (PDB ID: 6X41) in association with the drug candidates [68]. In this study, a 100‐ns simulation was conducted to monitor the RMSD of the Cα backbone of the CDTa protein (PDB ID: 6X41), concerning the drug candidates. Figure 3(a) reveals distinct fluctuations in protein–ligand compounds when complexed with 6X41. The glucosinolate complex exhibited a consistent state with an average RMSD value of 1.922 Å, closely resembling the apo protein (1.803 Å) and positive control ciprofloxacin (1.729 Å), implying that the binding of the ligand does not cause substantial rearrangements or distortions in the protein backbone. In contrast, indole‐3‐carbinol exhibited stable RMSD values until 30.75 ns, after which increased fluctuations suggested decreased stability, with an average RMSD of 2.28 Å. This indicates that indole‐3‐carbinol binding caused greater structural variations compared to glucosinolate, which is possibly due to the ligand causing localized conformational changes in the protein or weaker binding interactions [69]. On the other hand, the control complexes ciprofloxacin+ 6X41 remain almost stable during the simulation period.

Although the RMSD profile of the indole‐3‐carbinol complex shows larger deviations, this does not indicate inadequate equilibration. Rather, it reflects increased protein–ligand flexibility associated with weaker binding interactions.

### 3.4. Analysis of Radius of Gyration (Rg)

The radius of gyration (Rg) is the measurement of the RMS radial distance between the target protein’s center of mass and its terminals [70]. This analysis offers insights into the compactness of the protein–ligand complex by evaluating the protein’s rigidity and flexibility in response to specific ligands [71]. The glucosinolate complex remained stable with an average value of 24.119 Å during the 100‐ns simulation period, indicating a compact structure similar to the apo protein, and control ciprofloxacin, which also remained stable with an average value of 24.0 and 23.99 Å, respectively. Indole‐3‐carbinol showed minor fluctuations between 40 and 80 ns, with an average Rg of 24.28 Å, suggesting slightly less compactness. However, these slight fluctuations do not indicate the degree of conformational change induced by the binding of indole‐3‐carbinol to 6X41. The overall Rg analysis indicates that all complexes maintained a generally compact structure, although the glucosinolate complex was more stable than indole‐3‐carbinol (Figure 3(b)).

### 3.5. SASA

SASA measures the exposure of a protein to solvent, which can indicate conformational changes [72]. Over the 100‐ns simulation, the SASA profiles for the glucosinolate and indole‐3‐carbinol complexes with CDTa (PDB ID: 6X41) were stable, with mean values of 20,011.41 and 20,178.49 Å^2^, respectively. These values were similar to the apo protein’s SASA (19,751.86 Å^2^), indicating that neither ligand caused significant changes in the protein’s solvent exposure or overall conformation. The control complex ciprofloxacin + 6X41 also showed a stable SASA profile (20,210.46 Å^2^) until 80 ns, after which it exhibited a slight increase. Overall, the consistent SASA values across the complexes suggest that the binding of these ligands has a minimal impact on the protein’s structure or its exposure to the solvent when compared to the unbound state (Figure 4(a)).

FIGURE 4Analysis of (a) SASA and (b) hydrogen bond of complexes between indolylmethyl glucosinolate, indole‐3‐carbinol, and target protein of *Clostridioides difficile* toxin CDTa (PDB: 6X41) at 100 ns molecular dynamics simulation.

**Figure:**
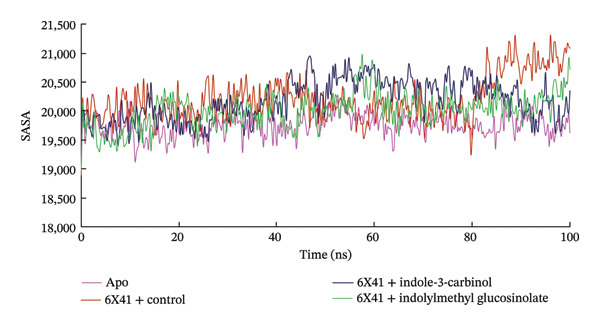
(a)

**Figure:**
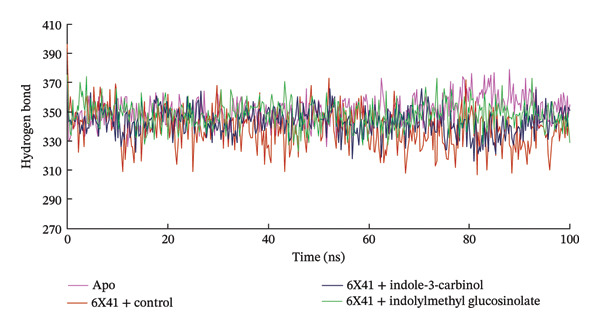
(b)

### 3.6. Analysis of Hydrogen Bond

Hydrogen bonding is among the key interactions that play a vital role in stabilizing the protein–ligand complex [73]. Figure 4(b) shows the number of hydrogen bonds formed between the selected ligands and CDTa (PDB ID: 6X41) over a 100‐ns simulation. Glucosinolate and indole‐3‐carbinol maintained stable hydrogen bond profiles throughout the 100‐ns simulation, like those observed in the apo protein and the control, ciprofloxacin. On average, glucosinolate, indole‐3‐carbinol, the apo protein, and ciprofloxacin formed 347.83, 342.84, 350.80, and 339.01 hydrogen bonds, respectively, suggesting strong and consistent binding interactions (Figure 4(b)). These stable hydrogen bond patterns, similar to those in the apo protein, and control indicate that both ligands maintain dynamic yet stable interactions with the protein, pointing to their potential inhibitory properties.

### 3.7. RMSF

The RMSF displays the flexibility and mobility of each amino acid residue in the apo protein and ligand‐6X41 complexes. A significant change in the RMSF value over 3 Å is considered significant and radically impacts the flexibility of amino acid residues [74]. The RMSF profile depicted in Figure 5(a) shows the flexibility of each amino acid residue in the 6X41‐ligand complexes. Based on the trajectory analysis, it is evident that both complexes exhibited similar fluctuations when bound to 6X41. Specifically, common fluctuations were observed for both indolylmethyl glucosinolate and indole‐3‐carbinolcomplexes during the initiation of the simulation analysis at amino acid residues THR1, THR2, TYR3, LYS4, ALA5, PRO6, and ILE7. The observed fluctuations at specific amino acid residues provide insights into the stability and structural changes induced by ligand binding. The closer resemblance of indole‐3‐carbinol to the apo protein state and positive control ciprofloxacin suggests a potentially more native‐like binding mode for both target proteins, which could be of significance in drug design and optimization efforts.

FIGURE 5The (a) RMSF and (b) MM–PBSA binding free energy of complexes at the 100‐ns simulation period.

**Figure:**
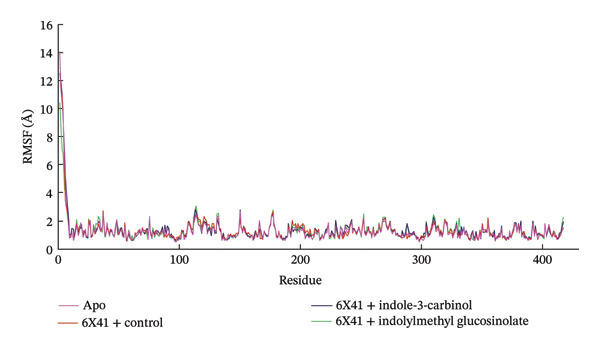
(a)

**Figure:**
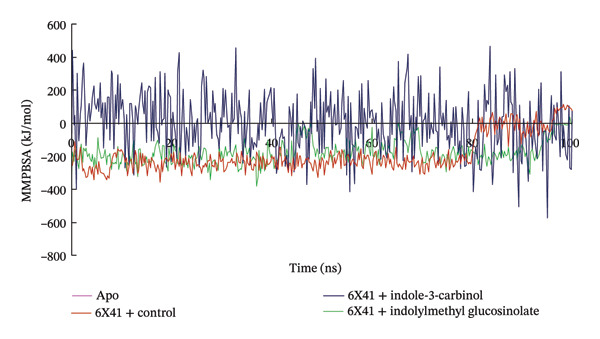
(b)

In the current study, the RMSD and RMSF analyses provide complementary insights into the dynamic behavior of the protein–ligand complexes. While the RMSF plot (Figure 5(a)) demonstrates relatively low atomic‐level fluctuations across the protein, indicating local stability, the RMSD plot (Figure 3(a)) shows notable global deviations in the overall structure. This can be attributed to rigid‐body motions or domain shifts, which cause significant structural rearrangements without affecting the local flexibility of individual residues. Therefore, the high RMSD values observed in certain complexes, such as with indole‐3‐carbinol, reflect these global conformational changes rather than local fluctuations, as captured by the RMSF. The key to resolving the apparent discrepancy lies in the distinction between local and global motions. The protein–ligand complex may experience global conformational changes (such as rotation, translation, or hinge‐like motions of domains) that result in a high RMSD, while the local atomic‐level flexibility remains low, leading to a flat RMSF. This is especially common in systems where the overall shape or orientation of the protein changes, but the internal secondary structures remain stable.

### 3.8. Analysis of MM–PBSA

The binding free energy of the docked complexes was calculated using the prime MM–PBSA method integrated within the YASARA modeling software suite. Figure 5(b) portrays the relative free binding energies of 6X41+ indolylmethyl glucosinolate, and 6X41+ indole‐3‐carbinol complexes were −176.14 and 4.03 kJ/mol. The significantly negative MM–PBSA value for the 6X41+ ciprofloxacin complex (−189.78 kJ/mol) and 6X41+ indolylmethyl glucosinolate indicates a strong binding affinity between the protein and ciprofloxacin, suggesting a stable and energetically favorable interaction that effectively binds to its target proteins. This suggests that indolylmethyl glucosinolate has a moderate affinity for the 6X41 protein, which could be relevant for their biological function. On the other hand, the 6X41+ indole‐3‐carbinol complex showed a slightly positive MM–PBSA value (4.03 kJ/mol), indicating an unfavorable or weak binding interaction. This suggests that indole‐3‐carbinol does not bind effectively to the 6X41 protein under the conditions of the simulation, which may imply limited biological relevance or activity in this context.

### 3.9. Analysis of Superimposition

The MD simulation snapshot of complexes of apo protein, indolylmethyl glucosinolate, indole‐3‐carbinol, and control ciprofloxacin at 0, 25, 50, 75, and 100 ns is shown in Figure 6. Based on these findings, the apo protein exhibited minimal structural changes at 0, 25, and 100 ns, although slight alterations were observed at 50 and 75 ns (Figure 6(a)). The compound indolylmethyl glucosinolate remained consistently positioned within the binding pocket of the target protein 6X41 throughout the simulation. The complexes exhibited high structural stability from 0 to 50 ns with no significant structural changes observed during simulation period (Figure 6(b)). However, variations in ligand–protein interactions were detected at 50, 75, and 100 ns compared with the interaction patterns at 0 and 25 ns (Figure 6(b)). Despite these slight fluctuations, both compounds maintained continuous interactions with the target protein throughout the entire simulation period, indicating stable binding and sustained complex integrity. Similarly, indole‐3‐carbinol demonstrated strong stability with the target protein at 0, 25, and 100 ns of the simulation (Figure 6(c)). The protein–ligand interaction patterns observed at 0 and 100 ns were almost identical, indicating restoration and maintenance of the binding conformation. However, at 50 and 75 ns, indole‐3‐carbinol temporarily dissociated from the binding pocket and showed no detectable interactions with the target protein during those intervals (Figure 6(c)). Despite this transient separation, the ligand re‐established interactions with the protein by 100 ns, suggesting a reversible binding behavior during the simulation period. Likewise, the target protein is sustained by control ciprofloxacin; nevertheless, minor structural alterations of the protein were noted, especially at 50 and 75 ns (Figure 6(d)). Interestingly, the control compound ciprofloxacin showed almost identical interaction patterns with the target protein at 0, 25, and 100 ns, indicating minimal conformational variation and strong binding stability across the simulation timeline (Figure 6(d)). Overall, the superimposition results indicate that all compounds formed a stable and strong interaction with the target protein throughout the simulation compared to the apo and control despite slight instability at 50 and 75 ns of indole‐3‐carbinol.

FIGURE 6Molecular dynamic simulation snapshot of (a) apo protein, and complexes between 6X41 with (b) indolylmethyl glucosinolate, (c) indole‐3‐carbinol, and (d) ciprofloxacin at 0, 25, 50, 75, and 100 ns. Here, in the superimposition panel, all snapshots are aligned to a common surface of apo protein, where pest, blue, violet, white, and purple colors represent the 0‐, 25‐, 50‐, 75‐, and 100‐ns snapshots.

**Figure:**
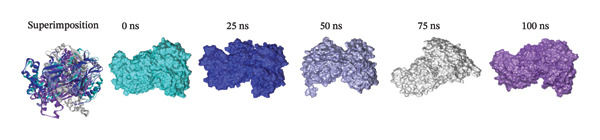
(a)

**Figure:**
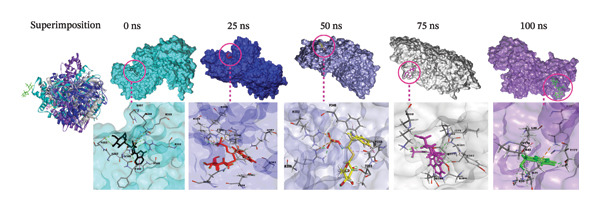
(b)

**Figure:**
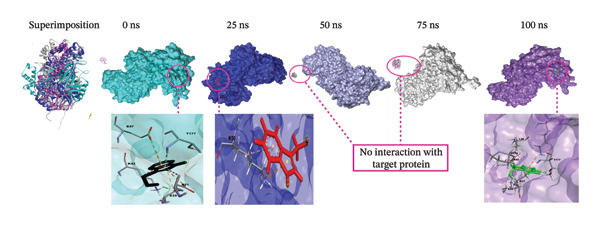
(c)

**Figure:**
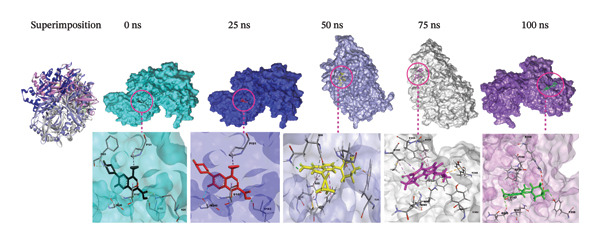
(d)

### 3.10. Analysis of PCA

The PCA results for the protein–ligand complexes with protein 6x41 reveal distinct patterns of structural variability across different systems (Figure 7). The apo form showed the highest conformational fluctuation, with the first two principal components (PC1 and PC2) accounting for 45.27% and 14.84% of the total variance, respectively, indicating significant flexibility in the absence of any ligand (Figure 7(a)). In contrast, the control complex exhibited more constrained dynamics, with PC1 and PC2 contributing 20.25% and 12.08%, suggesting reduced mobility upon ligand binding (Figure 7(b)). Among the test compounds, the glucosinolate complex displayed moderate flexibility (PC1: 26.33%, PC2: 19.19%) (Figure 7(c)), while the indole‐3‐carbinol complex showed a substantial variance in PC1 (36.64%) but less in PC2 (12.44%), reflecting a more dominant unidirectional conformational shift (Figure 7(d)). Overall, these results suggest that ligand binding differentially affects the dynamic behavior of protein 6x41, with the apo form being the most flexible and ligand‐bound forms exhibiting more restricted yet distinct motions. Additionally, the PCA results are consistent with the docking and in vitro inhibition data. They suggest that ligand binding (especially indole‐3‐carbinol) stabilizes the protein and restricts its conformational freedom, a typical sign of functional inhibition.

FIGURE 7Principal component analysis (PCA) of protein 6x41 in different states to evaluate conformational dynamics. (a) Apo form of the protein showing high structural flexibility. (b) Protein in complex with the control ligand demonstrating reduced motion compared to the apo form. (c) Indolylmethyl glucosinolate–bound complex indicating moderate stabilization of the protein structure. (d) Indole‐3‐carbinol‐bound complex displaying a distinct conformational restriction, suggesting a strong interaction and structural stabilization. PCA was performed on 100‐ns MD trajectories, and the first two principal components (PC1 and PC2) are plotted to illustrate the dominant motions in each system.

**Figure:**
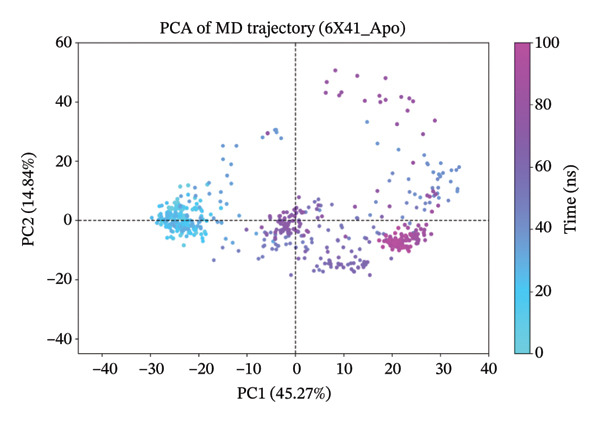
(a)

**Figure:**
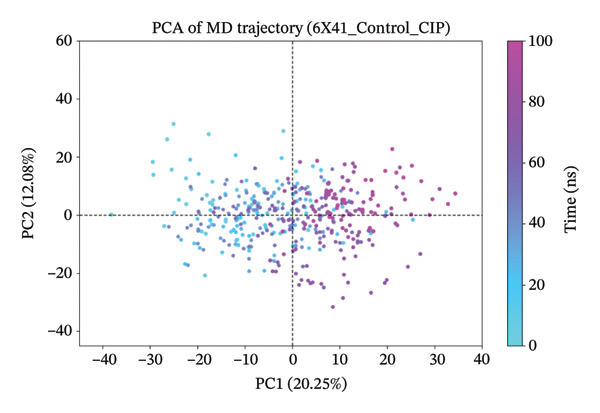
(b)

**Figure:**
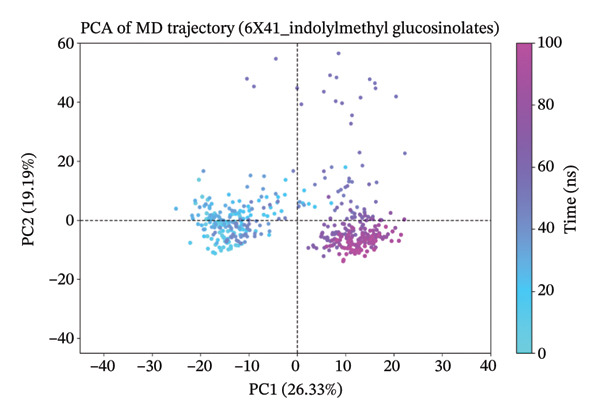
(c)

**Figure:**
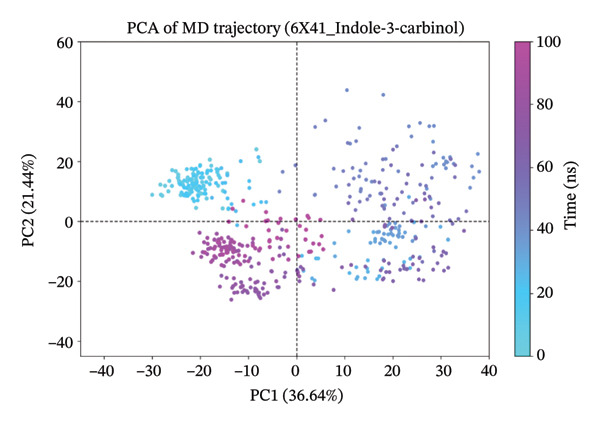
(d)

### 3.11. DCCM

The DCCM plots for a protein in different states with Apo (Figure 8(a)), control (Figure 8(b)), indolylmethyl glucosinolate (Figure 8(c)), and indole‐3‐carbinol (Figure 8(d)) complexes illustrate the correlated motions between residue pairs within the protein, with red indicating positive correlation (residues moving in the same direction), blue indicating negative correlation (residues moving in opposite directions), and white/light shades indicating no significant correlation (Figure 8). The intensity of the color corresponds to the correlation coefficient, ranging from −1.00 (strong negative) to 1.00 (strong positive). Comparing the Apo and control states, distinct differences in correlated motions are observed, particularly in the larger “control” system (Figure 8(b)), which shows more extensive regions of strong positive correlations (deep red blocks) and pronounced negative correlations (deep blue blocks) across a wider range of residue indices (up to ∼800 compared to ∼400 for Apo). In the presence of ligands, both the indolylmethyl glucosinolate and indole‐3‐carbinol complexes exhibit altered correlation patterns compared to the Apo state, suggesting that ligand binding induces changes in the protein’s dynamics. For instance, the indolylmethyl glucosinolate complex shows some areas of increased positive correlation around residue indices 50–100 and 200–250 compared to the Apo state, while the indole‐3‐carbinol complex displays a more localized reduction in some positive correlations seen in the Apo state, particularly around the 150–200 residue index range. These changes in correlated motions provide insights into how ligand binding affects flexibility and allosteric communication within the protein.

FIGURE 8Dynamic cross‐correlation matrix (DCCM) of protein in different states. The DCCM matrices depict the correlated motions between protein residue pairs. Red indicates positively correlated motions, blue indicates negatively correlated motions, and white/light shades indicate no significant correlation. The intensity of the color represents the correlation coefficient, ranging from −1.00 (strong negative) to 1.00 (strong positive). (a) Apo protein, (b) control, (c) protein in complex with indolylmethyl glucosinolate, and (d) protein in complex with indole‐3‐carbinol.

**Figure:**
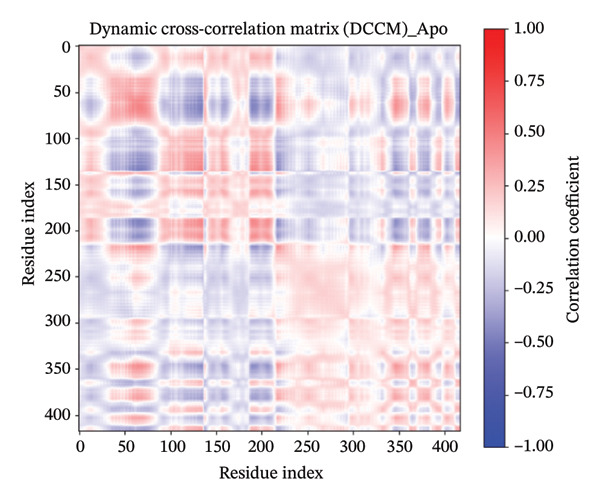
(a)

**Figure:**
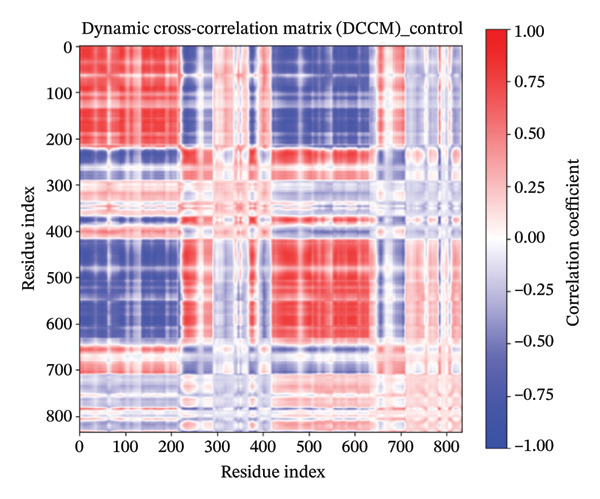
(b)

**Figure:**
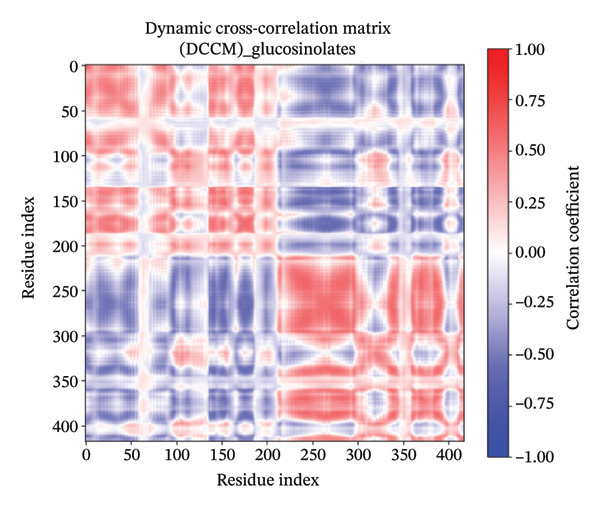
(c)

**Figure:**
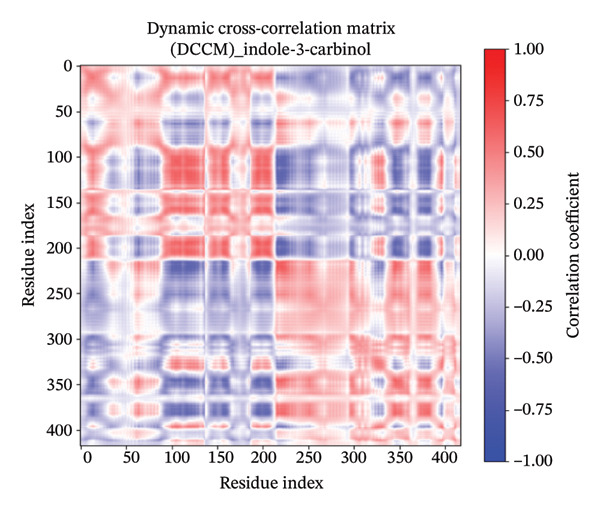
(d)

### 3.12. *In Vitro* Antibacterial Activity

The antibacterial activity of indolylmethyl glucosinolate and indole‐3‐carbinol against *C. difficile*, a bacterium implicated in CDI, was evaluated *in vitro* using the disc diffusion method. Table 2 presents the MIC and MBC values of two compounds—indolylmethyl glucosinolate and indole‐3‐carbinol—against *C. difficile*. The MIC represents the lowest concentration needed to inhibit visible bacterial growth, while the MBC indicates the concentration required to kill the bacteria. Indolylmethyl glucosinolate demonstrated an MIC of 10.33 ± 0.72 μg/mL (*p* < 0.05) and an MBC of 23.33 ± 1.36 μg/mL (*p* < 0.05), showing slightly greater antibacterial potency compared to indole‐3‐carbinol, which had an MIC of 11.33 ± 0.27 μg/mL (*p* < 0.05) and an MBC of 26.67 ± 3.60 μg/mL (*p* < 0.05). These results suggest that both compounds possess notable antimicrobial activity against *C. difficile*, with indolylmethyl glucosinolate being marginally more effective.

**TABLE 2:** MIC values quercetin and standard ciprofloxacin against *Clostridioides difficile*.

Compounds	MIC (*μ*g/mL)	MBC (*μ*g/mL)
Indolylmethyl glucosinolate	10.33^a^ ± 0.72	23.33^a^ ± 1.36
Indole‐3‐carbinol	11.33^a^ ± 0.27	26.67^a^ ± 3.60

*Note:* Where data are presented as mean ± SE, and *p* < 0.05 in comparison with the positive and negative control groups according to Duncan’s multiple range test and one‐way ANOVA. Values marked with different superscript letters (e.g., “a”) indicate statistically significant differences among groups. Values sharing the same letter are not significantly different, while those with different letters differ significantly at *p* < 0.05 according to one‐way ANOVA followed by Duncan’s multiple range test.

**FIGURE 9:**
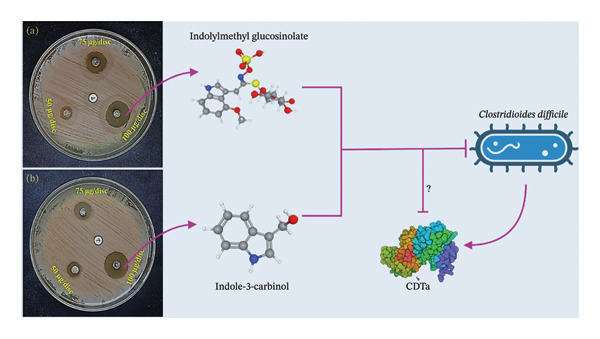
The antibacterial effects and possible mechanism of (a) indolylmethyl glucosinolate and (b) indole‐3‐carbinol against *Clostridioides difficile* at three distinct concentrations, with ciprofloxacin serving as the positive control.

Both compounds exhibited varying degrees of inhibitory effects against *C. difficile*, as indicated by the clear zones around the discs on the agar plates (Figure 9 and Table 3). At a concentration of 50 μg/disc, indolylmethyl glucosinolate produced an average inhibition zone of 11.33 ± 0.33 mm (*p* < 0.05), which increased to 18 ± 0.57 mm (*p* < 0.05) at 75 μg/disc and reached 23.33 ± 0.67 mm (*p* < 0.05) at 100 μg/disc. Likewise, indole‐3‐carbinol showed mean inhibition zones of 13.67 ± 0.33 mm, 16.16 ± 0.16 mm, and 22.67 ± 0.33 mm (*p* < 0.05) at concentrations of 50, 75, and 100 μg/disc, respectively. These results indicate that both compounds possess intrinsic antibacterial activity against *C. difficile*, independent of their predicted interaction with the CDTa toxin subunit. In comparison, the standard antibiotic ciprofloxacin did not show any inhibition at a concentration of 5 μg/disc, indicating that *C. difficile* is resistant to this antibiotic. This resistance aligns with previous findings that have demonstrated the antibacterial efficacy of *B. oleracea* extracts against various *Clostridium* species, although no prior studies have specifically investigated their effects on *C. difficile* [75, 76]. The difference in concentrations (5 μg/disc for ciprofloxacin versus 50, 75, and 100 μg/disc for indolylmethyl glucosinolate and indole‐3‐carbinol) is attributed to ciprofloxacin’s established potency as a broad‐spectrum antibiotic, effective even at lower doses. While ciprofloxacin typically serves as a reliable positive control, the resistance shown by *C. difficile* in this study highlights its potential limitations. Although ciprofloxacin demonstrated stable binding to CDTa in docking and MD simulations, this interaction did not translate into observable antibacterial activity in vitro. This apparent discrepancy is likely explained by the intrinsic fluoroquinolone resistance of the *C. difficile* strain used in this study, as well as the fact that ciprofloxacin’s primary antibacterial mechanism targets DNA gyrase and topoisomerase IV rather than toxin‐mediated pathways. Therefore, binding to CDTa alone is unlikely to be sufficient to inhibit bacterial growth in resistant strains, suggesting that the lack of activity reflects strain‐specific resistance rather than assay insensitivity. Conversely, the higher concentrations of indolylmethyl glucosinolate and indole‐3‐carbinol were necessary to assess their antibacterial potential due to their novelty as antimicrobial agents. These compounds, being naturally derived and less studied than conventional antibiotics like ciprofloxacin, warranted the use of higher concentrations to fully evaluate their inhibitory properties.

**TABLE 3:** The inhibition zones for *Clostridium difficile* at different concentrations of indolylmethyl glucosinolate and indole‐3‐carbinol, with ciprofloxacin serving as the control.

Compounds	Zone of inhibition (mean ± SE)			
	50 *μ*g/disc	75 *μ*g/disc	100 *μ*g/disc	CIP
Indolylmethyl glucosinolate	11.33^a^ ± 0.33	18^b^ ± 0.57	23.33^c^ ± 0.67	0
Indole‐3‐carbinol	13.67^a^ ± 0.33	16.16^a^ ± 0.16	22.67^b^ ± 0.33	0

*Note:* Values marked with different superscript letters (e.g., “a”, “b”, “c”) indicate statistically significant differences among groups. Values sharing the same letter are not significantly different, while those with different letters differ significantly at *p* < 0.05 according to one‐way ANOVA followed by Duncan’s multiple range test.

Indolylmethyl glucosinolate may play a role in antibiosis owing to its antibacterial activity, as supported by numerous previous studies [63, 77–81]. Conversely, the antibacterial effectiveness of indole‐3‐carbinol may have a significant impact on inhibiting bacterial growth, especially against *C. difficile* [64, 82, 83]. The enzyme myrosinase facilitates the hydrolysis of glucosinolates, producing unique chemicals that affect the growth of different microbes. [84]. Glucosinolates have a cytotoxic impact on *C. difficile* and can slow the growth of various harmful bacteria that create biofilms [85]. Indole‐3‐carbinol has been shown to inhibit bacterial efflux pumps, biofilm formation, FtsZ activity, and the pyruvate kinase activity of MRSA [86]. Considering the findings presented above, both indolylmethyl glucosinolate and indole‐3‐carbinol stand out as promising templates for the development of novel antibacterial agents, offering substantial potential for future research and practical applications.

While the computational analyses focused on the interaction of the selected compounds with the CDTa subunit of the binary toxin, it is important to distinguish toxin inhibition from bacterial growth suppression. CDTa is a secreted virulence factor primarily responsible for host cell cytotoxicity and is not essential for bacterial viability. Therefore, the growth inhibition observed in the antibacterial assays likely reflects additional mechanisms unrelated to CDTa targeting, such as interference with bacterial membrane integrity or metabolic processes. In this context, CDTa engagement is proposed to represent a complementary antivirulence mechanism that may attenuate toxin‐mediated pathogenicity rather than directly impair bacterial proliferation. This distinction is further supported by the observation that ciprofloxacin exhibited predicted interaction with CDTa in silico but did not inhibit bacterial growth, highlighting that CDTa binding alone is insufficient to affect *C. difficile* viability. Although toxin‐specific functional or secretion assays are required to experimentally validate antivirulence effects, the present findings suggest that the studied compounds may exert a dual functional profile combining antibacterial and antivirulence properties.

### 3.13. ADMET Analysis

To understand the pharmacokinetic profile and possible detrimental consequences of drugs, it is essential to evaluate their ADMET characteristics during medication development [87, 88]. Table 4 shows the results of the ADMET profile estimations performed using computational methods on the SwissADME and pkCSM platforms for indolylmethyl glucosinolate and indole‐3‐carbinol, respectively. The profiles were focused on the following: absorption, distribution, metabolism, excretion, and toxicity. Many parameters were used to assess absorption, including water solubility, CaCO_2_ cell permeability, and the human intestinal absorption (HIA) percentage. The water solubility LogSw for indolylmethyl glucosinolate was −2.683, and for indole‐3‐carbinol, it was −1.874. Because compounds with LogSw values below −6 are usually considered poorly soluble, these data show reasonable solubility [89]. When comparing indole‐3‐carbinol (1.586) to indolylmethyl glucosinolate (−0.848), the latter showed significantly poorer permeability with respect to CaCO_2_. With an estimated HIA value of 92.085% for indole‐3‐carbinol and 5.082% for indolylmethyl glucosinolate, indole‐3‐carbinol has a far higher potential for absorption in the human intestine.

**TABLE 4:** ADMET predictions for indolylmethyl glucosinolate and indole‐3‐carbinol, obtained through the SwissADME and PKCSM tools, showing that both compounds exhibit overall favorable drug‐like properties.

Parameters		Molecules	
		Indolylmethyl glucosinolate	Indole‐3‐carbinol
Absorption	Water solubility	−2.683	−1.874
	CaCO_2_ permeability	−0.848	1.586
	Intestinal absorption (human) (%)	Low (5.082%)	High (92.085%)

Distribution	VDss (human) (log L/kg)	−0.281	0.212
	BBB permeability	−1.54	0.439
	CNS permeability	−4.048	−2.17

Metabolism	CYP1A2 inhibitor	No	Yes
	CYP2D6 inhibitor	No	No
	P‐Glycoprotein I inhibitor	No	No
	P‐Glycoprotein II inhibitor	No	No

Excretion	Total clearance	0.375	0.515
	Renal OCT2 substrate	No	No

Toxicity	AMES toxicity	No	No
	Max. tolerated dose (human) (log mg/kg/day)	0.947	0.163
	hERG I inhibitor	No	No
	hERG II inhibitor	No	No
	Oral rat acute toxicity (LD_50_) (mol/kg)	2.537	2.39
	Oral rat chronic toxicity (LOAEL) (log mg/kg_bw/day)	3.72	1.77
	Hepatotoxicity	Yes	No
	Skin sensitization	No	Yes

The ability of indolylmethyl glucosinolate and indole‐3‐carbinol to inhibit important cytochrome P450 (CYP) enzymes, such as CYP1A2, CYP2D6, and P‐glycoprotein I and II, was used to assess their metabolic behavior. There is no concern that glucosinolates may cause metabolic interference or toxicity because they do not inhibit CYP1A2 or CYP2D6. This suggests that indolylmethyl glucosinolate has an advantageous metabolic profile, which may lessen the likelihood of medication interactions.

Additionally, it was discovered that indolylmethyl glucosinolate did not inhibit P‐glycoprotein I or II, suggesting that they probably will not interfere with drug efflux mechanisms. A good pharmacokinetic profile with lower risks of pharmacokinetic interactions is supported by the limited impact on the absorption and distribution of other drugs that rely on P‐glycoprotein transport. There may be metabolic interactions or toxicity concerns associated with this route due to indole‐3‐carbinol’s status as an inhibitor of the CYP1A2 enzyme. Thus, it is advised to use caution when administering indole‐3‐carbinol with medications that are processed by CYP1A2. It is highly unlikely that indole‐3‐carbinol will impact drug efflux or the pharmacokinetics of coadministered P‐glycoprotein substrates, as it does not inhibit P‐glycoprotein I or II, just as indolylmethyl glucosinolate. Indolylmethyl glucosinolate and indole‐3‐carbinol are both efficiently eliminated from the body at rates of 0.375 and 0.515 log mL/min/kg, respectively, suggesting that their accumulation and the potentially detrimental effects it entails are less likely. It is also not expected that the renal OCT2 transport protein will bind to either of these drugs. Indolylmethyl glucosinolate and indole‐3‐carbinol exhibit similar patterns of toxicity across a wide range of parameters.

Since neither compound is linked to AMES toxicity, it cannot be said that it causes mutations. The maximum tolerated dose (MTD) for indolylmethyl glucosinolate was 0.947 log mg/kg/day, which is greater than the MTD for indole‐3‐carbinol at 0.163 log mg/kg/day. This suggests that indolylmethyl glucosinolate may have better human tolerance. Neither chemical is expected to block hERG I or II channels; therefore, there is no concern about cardiac toxicity. Indolylmethyl glucosinolate and indole‐3‐carbinol both have equivalent acute toxicity levels in rats, with LD_50_ values of 2.53 mol/kg and 2.39 mol/kg, respectively, indicating that they have similar short‐term toxicological profiles. On the other hand, indole‐3‐carbinol has a lower potential for chronic toxicity (1.77 log mg/kg/bw/day) than indolylmethyl glucosinolate (3.72 log mg/kg/bw/day), suggesting that the two may differ in terms of long‐term safety. Indolylmethyl glucosinolate is anticipated to induce hepatotoxicity, in contrast to indole‐3‐carbinol, which is not anticipated to do so. Based on prior research and established standards, Table 4 details the evaluation of the pharmacokinetic characteristics and conformity with Lipinski’s rule for both substances [90–92].

This study builds upon our earlier investigation on derivatives from *B. oleracea* against bacterial toxins [93] with a novel focus on the binary toxin of *C. difficile*. The molecular weight of indolylmethyl glucosinolate is 448.47 Da, and they include 7 rotatable bonds, 10 hydrogen bond acceptors, and 6 hydrogen bond donors, giving them a total polar surface area (TPSA) of 215.58 Å^2^. While the majority of the properties adhere to Lipinski’s rule of five, there are a little too many hydrogen bond donors and acceptors (over 5 and 10 donors, respectively). However, indole‐3‐carbinol has properties that are within Lipinski’s acceptable range, such as a TPSA of 36.02 Å^2^, one rotatable bond, one hydrogen bond acceptor, and two hydrogen bond donors, and a molecular weight of 147.17 Da. Indolylmethyl glucosinolate and indole‐3‐carbinol both fall within the ideal 0‐3 range for lipophilicity (LogPo/w), suggesting adequate oral bioavailability. The indole‐3‐carbinol’s value is 1.51, whereas indolylmethyl glucosinolate varies from 0.46 to 1.51. Indolylmethyl glucosinolate may also have trouble being absorbed orally because their estimated bioavailability score was only 0.11, compared to 0.55 for indole‐3‐carbinol (Table 5) [93].

**TABLE 5:** The predicted outcomes for Lipinski’s rule and the pharmacokinetic characteristics of indolylmethyl glucosinolate and indole‐3‐carbinol, based on analysis from the SwissADME server.

PubChem ID	6537198	3712
Molecule name	Indolylmethyl glucosinolate	Indole‐3‐carbinol
Formula	C_16_H_20_N_2_O_9_S_2_	C_9_H_9_NO
Molecular weight	448.47	147.17
Number of rotatable bonds	7	1
Number of H‐bond acceptors	10	1
Number of H‐bond donors	6	2
TPSA	215.58	36.02
Log Po/w (WLOGP)	0.46	1.51
Lipinski violations	2violations: NorO > 10, NHorOH > 5	0
Bioavailability score	0.11	0.55

*Note:* The results suggest that both compounds possess favorable drug‐like attributes overall. (Partially adapted from our previous work on *Brassica oleracea*‐derived compounds targeting *E. coli* CdtB with permission and expanded for the current study on *C. difficile* binary toxin.) [93].

### 3.14. Limitation of the Study

While the present study provides mechanistic insights into ligand–CDTa interactions through molecular docking, MD simulations, and MM–PBSA calculations, we acknowledge that direct experimental confirmation of these interactions was not performed. Nevertheless, we complemented the computational analysis with in vitro antibacterial assays, which demonstrated growth‐inhibitory effects of indolylmethyl glucosinolate and indole‐3‐carbinol against *C. difficile*. Direct experimental validation using biophysical approaches such as microscale thermophoresis (MST), isothermal titration calorimetry (ITC), or NMR titration would further strengthen the evidence by providing quantitative binding affinities. Future studies should aim to experimentally validate the predicted ligand–CDTa interactions to fully characterize their antivirulence potential.

## 4. Conclusion

This study provides a detailed investigation into the interactions between selected bioactive compounds and the CDTa subunit of the *C. difficile* binary toxin (PDB ID: 6X41), employing MD simulations conducted over a 100‐ns time frame. The structural stability and binding affinity of ciprofloxacin, indole‐3‐carbinol, and indolylmethyl glucosinolate to the protein were evaluated using several metrics, such as RMSD, Rg, and SASA. Among the ligands examined, indolylmethyl glucosinolate demonstrated superior stability and a consistently compact conformation throughout the simulation period, indicating a strong and stable interaction with CDTa. Indole‐3‐carbinol also maintained favorable interactions, despite exhibiting minor conformational shifts that did not compromise protein structure. In parallel, in vitro antimicrobial assays revealed that indolylmethyl glucosinolate exhibited potent growth‐inhibitory activity against *C. difficile*, as supported by MIC and MBC values, indicating intrinsic antibacterial effects. Importantly, the predicted interaction with CDTa is proposed to represent a complementary antivirulence mechanism, potentially contributing to the attenuation of toxin‐mediated pathogenicity rather than directly accounting for bacterial growth inhibition. These findings provide valuable mechanistic insights into ligand–CDTa interactions while also highlighting the antibacterial properties of *B. oleracea*–derived compounds. Indolylmethyl glucosinolate emerges as a promising candidate for further exploration as a therapeutic agent against CDI, either as a standalone antibacterial compound or as an adjunct targeting toxin‐mediated virulence. Future studies focusing on pharmacokinetic profiling and in vivo validation will be essential to assess efficacy, safety, and translational potential.

**Nomenclature**
CDI: *Clostridioides Difficile* Infection
CDT: *Clostridioides Difficile* Transferase (Binary Toxin)
CDTa: Enzymatic Subunit a of CDT
MIC: Minimum Inhibitory Concentration
MBC: Minimum Bactericidal Concentration
ADMET: Absorption, Distribution, Metabolism, Excretion, and Toxicity
MMGBSA: Molecular Mechanics Generalized Born Surface Area
RMSD: Root‐Mean‐Square Deviation
RMSF: Root‐Mean‐Square Fluctuation
SASA: Solvent‐Accessible Surface Area
Rg: Radius of Gyration
PDB: Protein Data Bank
LB: Luria Bertani (Broth/Agar)
CFU: Colony Forming Units

## Author Contributions

Ariful Islam, Sumaiya Jahan Supti, and Faria Tasnim: conceptualization, data curation, formal analysis, investigation, visualization, methodology, validation, and writing–original draft and review and editing. Mst Naharina Nuryay, Nabida Tabassum, Md. Zahid Hasan, Md. Jan Sadur Rahman Moon, and Maysha Fahmeda Priota: data curation, formal analysis, visualization, validation, and review and editing. Taha Alqahtani, Magdi E. A. Zaki, and Subir Sarker: data curation, methodology, validation, funding acquisition, project administration, resources, and review and editing. Md. Eram Hosen: conceptualization, data curation, formal analysis, methodology, project administration, resources, supervision, validation, and review and editing.

## Funding

This study received no external funding.

Open access publishing facilitated by James Cook University, as part of the Wiley ‐ James Cook University agreement via the Council of Australasian University Librarians.

## Ethics Statement

The authors have nothing to report.

## Consent

The authors have nothing to report.

## Conflicts of Interest

The authors declare no conflicts of interest.

## Supporting Information

Additional supporting information can be found online in the Supporting Information section.

## Supporting information


**Supporting Information** Supporting Figure S1. Molecular docking interactions of the compound (a) glucosinolates and (b) indole‐3‐carbinol from *B. oleracea* with 6X41 protein of *C. difficile* during the MD simulation period; surface, and 2D view of compounds, where ciprofloxacin used as a positive control. Supporting Table S1. Interaction of the 51‐ligand molecule from *B. oleracea* against 6X41 protein of *C. difficile* mentioning binding energy, noncovalent interaction, interacting amino acids, bond types, and their distance, where ciprofloxacin used as a positive control. Supporting Table S2. Interaction of the ligand molecule glucosinolates and indole‐3‐carbinol against the 6X41 protein of *C. difficile* mentioning noncovalent interaction, interacting amino acids, bond types, and their distance at the MD simulation period.

## Data Availability

All data generated or analyzed during this study are included in this manuscript and its supporting file 1.
