# Supplementary material for: Mechanistic Insights Into the Inhibition of Clostridioides difficile Binary Toxin by Indolylmethyl Glucosinolate and Indole‐3‐Carbinol
Source: Scientifica (Cairo). 2026 May 18;2026:8115947. doi: 10.1155/sci5/8115947 (PMC13184179; doi:10.1155/sci5/8115947)

*Supplementary file 1*

**Mechanistic insights into the inhibition of *Clostridioides difficile* binary toxin by indolylmethyl glucosinolate and indole-3-carbinol**

Ariful Islam^1,#^, Sumaiya Jahan Supti^1,#^, Faria Tasnim^1,2^, Md. Zahid Hasan^3^, Mst Naharina Nuryay^3^, Nabida Tabassum^3^, Maysha Fahmeda Priota^3^, Md. Jan Sadur Rahman Moon^3^, Taha Alqahtani^4^, Magdi E. A. Zaki^4^, Subir Sarker^5’*^, Md. Eram Hosen^2, 5,*^

**Supplementary Table S1.** Interaction of the 51-ligand molecule from *Brassica oleracea* against 6X41 protein of *Clostridioides difficile* mentioning binding energy, non-covalent interaction, interacting amino acids, bond types and their distance. Where ciprofloxacin used as a positive control.

| **SL.** | **Complex** | **Binding energy (kcal/mole)** | **Amino acid residues** | **Bond types** | **Distance (Å)** |
| --- | --- | --- | --- | --- | --- |
| **1** | 6X41+ 6537198 | -9.1 | A:ASN262  A:ARG302  A:ARG302  A:ASN342  A:ASN263  A:ASN263  A:PHE343  A:ARG359  A:GLN307  A:PHE345  A:PHE345  A:GLU308 | H  H  H  H  H  H  H  H  C-H  P-P  P-P  AC | 2.15  2.21  2.75  2.52  2.28  2.96  2.48  5.34  3.51  4.00  4.71  5.31 |
| **2** | 6X41+ 3712 | -8.75 | A:ALA31  A:GLU35  A:GLU47  A:THR177  A:LYS43 | H  H  H  H  P-A | 1.85  4.77  4.92  3.04  5.00 |
| 3 | 6X41+5281167 | -5.2 | A:ARG303  A:PHE356  A:MET353 | H  Pi-Alkyl  Alkyl | 2.14  4.72  4.82 |
| 4 | 6X41+5318042 | -5.1 | A:MET353  A:ARG303  A:PHE345 | Alkyl  Alkyl  C-H | 4.45  4.27  3.52 |
| 5 | 6X41+520108 | -7.3 | A:ARG364  A:ASP402  A:LYS139  A:ASP141  A:ILE413 | Pi-Cation  Pi-Anion  H  H  Pi-Alkyl | 3.79  3.52  1.87  2.44  5.39 |
| 6 | 6X41+5324797 | -7.0 | A:ARG303  A:TYR301  A:ARG302  A:MET353 | H  H  H  Pi-Sulfur | 2.16  3.00  2.86  4.88 |
| 7 | 6X41+444972 | -7.98 | A:LYS37  A:ASP93  A:GLU30  A:ILE92 | H  H  H  C-H | 2.04  2.41  2.33  3.35 |
| 8 | 6X41+518838 | -5.78 | A:PHE356  A:ARG302  A:ARG303 | Pi-Alkyl  Pi-Alkyl  Pi-Alkyl | 3.90  4.58  4.53 |
| 9 | 6X41+526561 | -6.95 | A:ILE413  A:LYS139  A:ASP141  A:ASP402 | Alkyl  Pi-Alkyl  Pi-Anion  Pi-Anion | 5.00  4.70  4.81  3.43 |
| 10 | 6X41+351795 | -4.89 | A:PHE66  A:PHE66  A:LYS405 | Pi-Pi  Pi-Pi  H | 4.32  5.61  2.85 |
| 11 | 6X41+9888239 | -3.2 | A:LYS139  A:ASP402 | Unfavorable Acceptor-Acceptor  H | 2.98  2.19 |
| 12 | 6X41+5280450 | -8.1 | A:SER347  A:TYR301  A:PHE356  A:ALA355  A:MET353  A:PHE345 | C-H  H  Pi-Alkyl  Alkyl  Alkyl  Pi-Alkyl | 3.44  2.20  4.06  4.31  4.31  4.03 |
| 13 | 6X41+5280934 | -8.2 | A:SER347  A:PHE356  A:ALA383  A:TYR382  A:PHE345 | C-H  Pi-Alkyl  Pi-Alkyl  Pi-Alkyl  Pi-Alkyl | 3.58  4.38  4.39  5.27  3.96 |
| 14 | 6X41+656506 | -8.0 | A:ASP222  A:PHE220  A:GLU187  A:ARG84  A:LYS221 | H  H  Pi-Anion  Pi-Alkyl  H | 2.44  2.21  3.73  5.45  2.83 |
| 15 | 6X41+5280489 | -8.7 | A:ALA383  A:PHE356  A:TRP333  A:TYR340  A:TYR382 | Pi-Alkyl  Pi-Alkyl  Pi-Alkyl  Pi-Alkyl  Pi-Alkyl | 5.27  4.81  5.48  3.79  5.31 |
| 16 | 6X41+6920 | -7.8 | A:ASP141  A:ASP402  A: ILE413  A: ARG364 | Pi-A  Pi-A  Pi-Al  Pi-C | 4.38  4.12  4.97  4.41 |
| 17 | 6X41+12020 | -7.1 | A: LEU 46  A: LYS 50  A: THR 177  A: LEU 38 | Al  UDD  CH  Al | 5.34  2.09  2.04  5.11 |
| 18 | 6X41+8723 | -3.5 | A: ARG 303 A: PHE 356 | Al  Pi-Al | 4.37  4.90 |
| 19 | 6X41+6322 | -7.88 | A: LYS 106  A: ASP 402  A: ILE 413  A: GLN 61  A:ASP 141 | CH  CH  CH  PA  PA | 5.75  2.27  2.93  3.64  5.29 |
| 20 | 6X41+8078 | -3.1 | A: ARG 303 A: PHE 356 | Al  Pi-S | 5.19  3.54 |
| 21 | 6X41+7938 | -7.90 | A: ALA 31  A: LEU 174  A: ILE 34  A:LEU 38  A:LEU 46 | CH  Al  Al  Al  Al | 3.42  5.12  5.07  4.90  5.12 |
| 22 | 6X41+7362 | -7.66 | A: ARG 12  A:PRO 9  A: ALA 8  A: ARG 33  A:ASP 93 | CH  Pi-Al  Pi-Al  Pi-C  CH | 4.57  5.44  5.11  4.12  2.42 |
| 23 | 6X41+7284 | -3.0 | A: ARG 303  A: PHE 35 | Al  Pi-A | 3.87  4.52 |
| 24 | 6X41+6782 | -6.81 | A: PHE 345 A: TYR 258 A: ARG 359 A: ARG 302 A: PHE 356 | Pi-S  Pi-T  CH  CH  Pi-S | 4.28  5.41  2.56  2.74  4.00 |
| 25 | 6X41+8091 | -6.0 | A: ASN 342  A: ASN 262  A:PHE 345  A: TYR 253 | CH  CH  CH  Pi-Al | 5.10  2.72  2.08  4.19 |
| 26 | 6X41+240 | -6.40 | A: ARG303  A: PHE345  A: THR346  A: MET353  A: PHE356 | H, Pi-Al  CH  Am-Pi  Pi-Sul  Pi-Pi | 2.15,5.15  3.45  4.91  4.75  4.32 |
| 27 | 6X41+311 | -7.12 | A: ARG302  A: ARG303  A: PHE345  A: SER347  A: PHE356 | H, UDD  UDD  H  H, CH  Pi-Si | 1.65, 2.65  2.43  1.89  2.84, 3.09  3.52 |
| 28 | 6X41+798 | -7.0 | A: ARG302  A: ARG303  A: PHE345  A: MET353 | Pi-Al  Pi-Al  CH  Pi-Al | 5.35  5.07  3.49  5.26 |
| 29 | 6X41+1068 | -7.15 | A: ALA8  A: PRO9  A: TRP26  A: ILE92  A: ASP93 | Al  Al  Pi-Al  Al  H | 4.50  4.53  4.96  5.22  2.88 |
| 30 | 6X41+1140 | -3.11 | A: TYR258  A: PHE345 | Pi-Pi T, Pi-Al  Pi-Pi S, Pi-Al | 4.91, 4.24  3.94, 3.92 |
| 31 | 6X41+2682 | -7.5 | A: TYR258  A: ARG303  A: PHE345  A: MET353  A: PHE356 | H, Pi-Al  Al  Pi-Al  Al  Pi-Al, Pi-Al, Pi-Al | 2.22, 2.45  4.30  3.76  5.05  3.81, 4.86, 5.12 |
| 32 | 6X41+3026 | -7.03 | A: TYR258  A: ARG302  A: PHE345  A: PHE356  A: ARG359 | Pi-Pi T  H, H  Pi-Pi S  Pi-Al  H | 5.07  2.47, 2.95  4.11  4.46  2.81 |
| 33 | 6X41+6184 | -3.14 | A: MET353  A: PHE356 | Al  Pi-Al | 4.82  4.90 |
| 34 | 6X41+6274 | -4.80 | A: ARG12  A: GLU30  A: ASN90  A: ASP93 | H  H  H, UDD  H | 4.73  2.65  1.03, 180  223 |
| 35 | 6X41+6306 | -4.68 | A: TYR6  A: PRO9  A: ASN90 | Pi-Al, Pi-Al  CH, Al  H, H, H | 4.45, 5.49  3.52, 4.18  2.21, 2.39, 2.58 |
| 36 | 6X41+12232 | -5.12 | A:TYR149  A:PRO151  A:PRO151  A:PHE101 | Alkyl  Alkyl  Pi-Alkyl  Pi-Alkyl | 4.47  4.03  4.32  5.06 |
| 37 | 6X41+12244 | -4.3 | A:ILE413  A:LYS106  A:ASP141 | Alkyl  H  H | 4.21  2.41  2.89 |
| 38 | 6X41+12372 | -4.90 | A:PHE345  A:PHE345  A:ASN342  A:ASN262 | Pi-Alkyl  Pi-Alkyl  H  H | 3.56  4.87  2.95  5.21 |
| 39 | 6X41+14286 | -5.5 | A:ASN141  A:LYS106  A:LYS139 | H  H  H | 2.35  2.08  1.89 |
| 40 | 6X41+14505 | -5.12 | A:MET353  A:ARG303  A:ARG303  A:ARG303 | Pi-Sulfur  H  H  Pi-Alkyl | 4.85  2.09  2.62  3.99 |
| 41 | 6X41+18554 | -5.67 | A:PHE356  A:ARG303  A:ARG303  A:MET353 | Alkyl  Alkyl  Pi-Alkyl  Pi-Sulfur | 3.90  4.19  4.00  4.83 |
| 42 | 6X41+19602 | -7.0 | A:THR346  A:SER347  A:PHE356  A:ARG303  A:PHE356 | Amaide-Pi- Stacked  Van der Waals  Pi-Pi Stacked  Pi-Alkyl  Pi-Alkyl | 4.99  4.99  4.08  4.82  5.45 |
| 43 | 6X41+19754 | -7.6 | A:ARG302  A:ARG303  A:ARG303  A:ARG303  A:PHE356 | Alkyl  Pi-Alkyl  Pi-Alkyl  Alkyl  Pi-Sigma | 4.64  4.05  4.42  4.41  3.76 |
| 44 | 6X41+20307 | -5.1 | A:PHE356  A:GLY349  A:ARG303 | Pi-Alkyl  C-H  H | 3.86  3.24  2.11 |
| 45 | 6X41+22201 | -7.34 | A:ARG364  A:ASP402  A:LYS139  A:ILE413  A:ILE413 | Pi-Cation  Pi-Anion  H  Alkyl  Pi-Alkyl | 3.86  3.46  1.96  5.17  4.69 |
| 46 | 6X41+30215 | -7.87 | A:ILE413  A:TYR65  A:ARG364  A:ASP141  A:LYS139 | Pi-Alkyl  Pi-Alkyl  Pi-Anion  Alkyl  H | 4.11  4.93  2.51  4.14  4.75 |
| 47 | 6X41+31252 | -7.6 | A:LYS139  A:ILE413  A:ASP141  A:ASP402 | Pi-Alkyl  Alkyl  Pi-Anion  Pi-Anion | 4.68  5.00  4.74  3.44 |
| 48 | 6X41+31260 | -3.44 | A:PHE356  A:ARG303 | Pi-Alkyl  H | 4.53  2.14 |
| 49 | 6X41+61653 | -7.15 | A:ARG364 A:ASP402 A:LYS139  A:LYS106 | Alkyl  Pi-Cation  Pi-Cation  Alkyl | 4.02  4.18  2.30  2.13 |
| 50 | 6X41+93320 | -7.21 | A:THR177 A:LEU46  A:LEU38  A:LYS43 | H  Alkyl  Alkyl  Alkyl | 2.25  4.83  5.33  5.30 |
| 51 | 6X41+123388 | -4.85 | A:ARG303  A:PHE356  A:MET353 | H  Pi-Alkyl  Pi-Sigma | 2.81  4.33  3.77 |

**Supplementary Table S2.** Interaction of the ligand molecule Glucosinolates and Indole-3-carbinol against 6X41 protein of *Clostridioides difficile* mentioning non-covalent interaction, interacting amino acids, bond types and their distance at MD simulation period.

| **Complex** | **Amino acid residues** | **Bond types** | **Distance (Å)** |
| --- | --- | --- | --- |
| 6X41+ Glucosinolates | A:ASN262  A:ARG302  A:ARG302  A:ASN342  A:ASN263  A:ASN263  A:PHE343  A:ARG359  A:GLN307  A:PHE345  A:PHE345  A:GLU308 | H  H  H  H  H  H  H  UPP  C-H  P-P  P-P  AC | 2.15  2.21  3.15  2.52  2.05  2.96  2.48  5.44  3.51  4.06  4.71  5.11 |
| 6X41+ Indole-3-carbinol | A:ALA31  A:GLU35  A:GLU47  A:THR177  A:LYS43 | H  H  H  CH  P-A | 1.85  5.00  4.70  3.14  5.00 |
| 6X41**+** Ciprofloxacin | A:LYS24  A:VAL182  A:PHE101  A:PRO151  A:SER147  A:ASP145 | H  H  CH  A  PS  HA | 2.27  1.95  3.45  4.54  3.80  3.17 |

**Supplementary Fig. S1.** Molecular docking interactions of the compound (a) Glucosinolates and (b) Indole-3-carbinol from *Brassica oleracea* with 6X41 protein of *Clostridioides difficile* during MD simulation period; surface, and 2D view of compounds. Where ciprofloxacin used as a positive control.


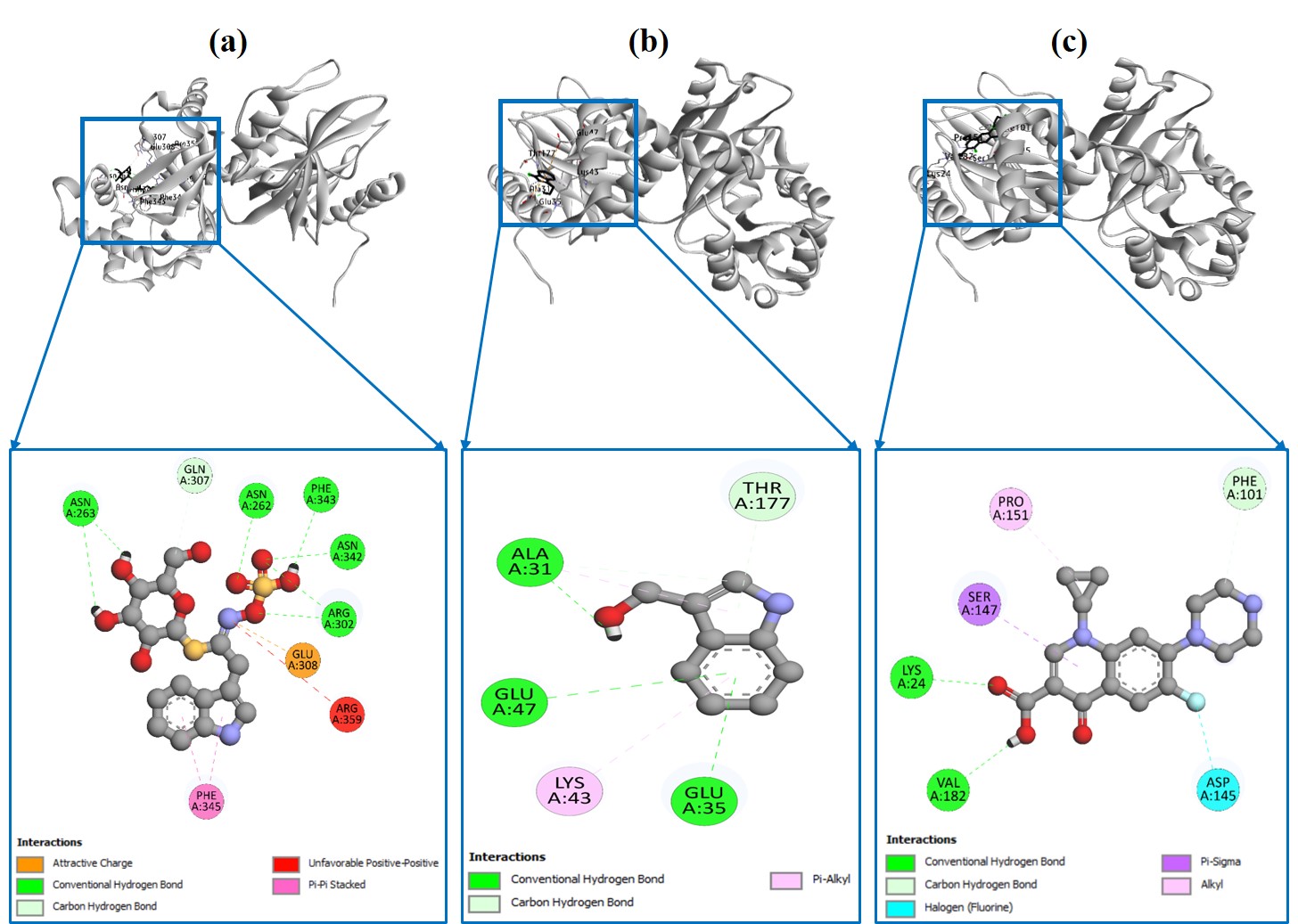

Supplement: Supplementary file 1 — Supporting Information Supporting Figure S1. Molecular docking interactions of the compound (a) glucosinolates and (b) indole‐3‐carbinol from B. oleracea with 6X41 protein of C. difficile during the MD simulation period; surface, and 2D view of compounds, where ciprofloxacin used as a positive control. Supporting Table S1. Interaction of the 51‐ligand molecule from B. oleracea against 6X41 protein of C. difficile mentioning binding energy, noncovalent interaction, interacting amino acids, bond types, and their distance, where ciprofloxacin used as a positive control. Supporting Table S2. Interaction of the ligand molecule glucosinolates and indole‐3‐carbinol against the 6X41 protein of C. difficile mentioning noncovalent interaction, interacting amino acids, bond types, and their distance at the MD simulation period. [file SCI5-2026-8115947-s001.docx]
